# Ninth International Dental Congress

**Published:** 1887-03

**Authors:** 


### Ninth International Medical Congress.

WASHINGTON, D. C., September 5, 1886.

President, N. S. Davis, M. D., LL. D.

Secretary General, John B. Hamilton, M. D., of U. S. A.

SECTION 17, DENTAL AND ORAL SURGERY.

President, Dr. J. Taft.

VICE-PRESIDENTS.

| | |
|---|---|
| Dr. W. W. Allport, 242 Wabash Avenue, | Chicago, Ill. |
| Dr. F. Abbott, 22 W. 40th St., - - | New York. |
| Dr. W. C. Barrett, 208 Franklin St., - | Buffalo, N. Y. |
| Dr. S. W. Dennis - - - - - | San Francisco, Cal. |
| Dr. C. L. Ford - - - - - | Ann Arbor, Mich. |
| Dr. W. H. Morgan, - - - - | Nashville, Tenn. |
| Dr. H. J. McKellops, 2630 Washington, Av., | St. Louis, Mo. |
| Dr. A. T. Metcalf, - - - - | Kalamazoo, Mich. |
| Dr. A. L. Northorp, 57 W. 49th St., - | New York City. |
| Dr. A. O. Rawls, - - - - | Lexington, Ky. |
| Dr. Joseph Richardson, - - - | Terre Haute, Ind. |
| Dr. C. W. Spalding, - - - - | St. Louis, Mo. |
| Dr. L. D. Shepard, 100 Boylston St., - | Boston, Mass. |
| Dr. James Truman, 3249 Chestnut St., | Philadelphia, Pa. |
| Dr. W. W. H. Thackston, - - - | Farmville, Va. |
| Dr. V. E. Turner, - - - - | Raleigh, N. C. |

SECRETARIES.

| | |
|---|---|
| Dr. E. A. Bogue, 29 East 20th St., - | New York City. |
| Dr. F. H. Rehwinkel, - - - | Chillicothe, O. |
| Dr. E. Brasseur, 6 Rue Mogador, - - | Paris, France. |
| Dr. Elof Fœrberg, - - - - | Stockholm. |
| Dr. Julius Parreidt, - - - - | Leipzig, Germany. |

FINANCE COMMITTEE OF THIS SECTION.

| | |
|---|---|
| Dr. J. W. White, Treas., 12th & Chestnut, | Philadelphia, Pa. |
| Dr. A. M. Dudley, Secy., - - - | Salem, Mass. |
| Dr. Edgar Palmer, - - - - | LaCrosse, Wis. |
| Dr. H. J. McKellops, 2630 Washington Av., | St. Louis, Mo. |
| Dr. L. D. Shepard, 100 Boylston St., - | Boston, Mass. |
| Dr. C. H. Winkler, - - - - | Augusta, Ga. |

Dr. W. W. Allport, 242 Wabash Av., - Chicago, Ill.
Dr. W. W. Walker, 67 W. 9th St., - New York

COMMITTEE ON OPERATIVE DENTISTRY AND ORAL SURGERY —CLINICS.

Dr. C. F. W. Bœdecker, 60 E. 58th St., New York.
Dr. J. A. Watling, - - - - Ypsilanti, Mich.
Dr. R. L. Cochran, - - - - Burlington, Iowa.
Dr. F. Abbott, 22 W. 40th St., - New York.
Dr. W. C. Wardlaw, - - - - Augusta, Ga.
Dr. J. D. Patterson, - - - Kansas City, Mo.

It will be the duty of this Committee to make ample provision for the work of this department, providing all needed facilities, such as chairs, engines, and all appliances; to provide patients and subjects for the Clinics; to confer with the operators, learn their needs, and supply them so far as possible.

There will be others added to this Committee as the work may require.

COMMITTEE ON PROSTHETIC DENTISTRY.

Dr. Geo. L. Field, - - - - Detroit, Mich.
Dr. H. B. Noble, - - - - Washington, D. C.
Dr. A. O. Hunt, . - - - - Iowa City, Iowa.
Dr. John Allen, - - - - New York, N. Y.
Dr. T. T. Moore, - - - - - Columbia, S. C.
Dr. N. W. Morrison, 1337 Washington Av., St. Louis, Mo.
Dr. R. B. Donaldson, - - - Washington, D. C.

It will be the duty of this Committee to provide facilities for the work of this department, and arrange for its efficient performance; and make such regulations as shall secure the greatest benefit from the demonstrations.

ORGANIZATION.

*The following rules and regulations have been adopted by the Ex-Committee for the guidance of the work of the Congress and of its sections.*

The Congress will consist of such members of the regular medical profession, as shall have registered and taken out their ticket of admission, and of such other scientific men, as the Executive Committee of the Congress shall deem it desirable to admit.

Books for registration of members will be ready on and after September 1st, 1887, and on each subsequent day during the session. Any member desiring registration prior to this time, may apply by letter to the Secretary General, and forward his dues with his full address, when a receipt will be returned.

The membership fee for residents of the United States will be (10.00) ten dollars; there will be no dues for members from other countries. Each member will be entitled to receive a copy of the transactions of the Congress when published by the Ex-Committee

The work of the various sections will be directed, by the President of the section, and the order will be published in a daily programme for each section.

Brief abstracts of papers to be read in the sections shall be forwarded to the secretaries of the proper section on or before April 30, 1887. These abstracts shall be treated as confidential communications. Papers relating to topics not included in the list of subjects proposed by the officers of the sections may be accepted after April 30th, 1887.

The officers of each section shall decide as to the acceptance of such proposed communications, and the time for their presentation.

The Ex-Committee cordially invites members of the regular Medical profession, and men eminent in the *Sciences, collateral to Medicine, in all countries*, to participate in person, or by papers, in the work of this great humanitarian assembly.

The attendance of medical students, and others interested in the work of the various sections, or in the general addresses delivered in Congress, will be permitted on the recommendation of the Secretary General, or the officers of a section, on their taking out of the registration committee a general ticket of admission fee, one dollar; such persons cannot take part in the proceedings.

All communications and questions relating to the special business of any section, must be addressed to the President, or one of the Secretaries of that section.

OFFICERS OF SECTIONS, AND THEIR DUTIES.

The officers of each section, including foreigners shall be a

*President*, not less than five *Vice Presidents*, four *Secretaries*, (two foreign) and not less than ten nor more than thirty, members of council.

*President.*—The President of each section, shall be its executive officer, who is solely responsible for the efficient work of his section. He shall nominate all persons to the Ex-Committee, for any office connected with his section. He shall select and regulate (by conference where desired, with the other officers of his section), all papers or questions for discussion and reject only after such conference, such papers and questions, as he may deem inadmissible to the transactions, or for presentation in his section. He shall preside at, and regulate the business of each meeting of his section, punctually at the hour named, making an opening address to the section, if he so desires. He may at any time adjourn the session of the section, when in his opinion sufficiently long, as when there are too many papers for the day, etc., etc. He shall strictly enforce Rule 8 of the preliminary Organization. When a paper is read, or discussion occurs in a foreign language, he shall resign the chair to a foreign officer of the same nationality as the language employed.

*Vice-Presidents.*—They shall assist the President in the performance of his duties at each meeting of the Section when requested by him, and shall take their seats on each side of the presiding officer. They shall aid the President in consultation on the value and character of all papers or discussions that are to be presented in the Section or the transactions.

*Secretaries.*—The four Secretaries of the Sections shall arrange among themselves, or at the request of the President of the Section, the order of their duties. They shall keep accurate records of the proceedings of each day in their Section, and make such daily report of it to the Congress, as the President of the Section shall direct; the foreign Secretaries acting for their own nationalities. At the close of the session they shall present all their Minutes, in good order, to the Secretary General for publication in the Transactions, if so desired by the Executive Committee.

*Members of Council.*—It is expected that the Members of the Council will aid in every way to make the Sessions efficient and instructive. They will also be expected to advise with the President on any matter pertaining to the work of the Section, and on questions which may arise in connection with the publication, in the transactions of papers read, or discussion had in the Section.

### International Medical Congress.—Trans-Atlantic Rates.

In an official notice from the Chairman of the Committee of Arrangements at Washington, he says that reliable arrangements have been made by which members wishing to attend the International Medical Congress in Washington September 5, 1887, can be accommodated by the following steamship lines at the liberally reduced rates mentioned, viz:

Red Star Line.—$100, Antwerp—New York and return.

Inman Line.—$100, Liverpool—New York and return.

Hamburg Line.—$90, Hamburg—New York and return.

Royal Netherland.—$80, Amsterdam—New York and return.

Since that notice we have received authentic information that the several lines named have consented to extend the same rates to the families of members, as the following letter shows:

WASHINGTON, D C., FEB. 14, 1887.

A. Y. P. GARNETT, M. D., *Chairman of the Committee of Arrangements of the International Medical Congress:*

*Dear Doctor*:—I am happy to inform you that, through the instrumentality of Mr. Edward F. Droop, agent in this city for the Lines, who has manifested so much interest in this matter, we have been able to secure from the Hamburg-American, the Red Star, and the Inman Lines the offer of the same reductions for the families of members of the Congress as those I have already reported for the members themselves. Very Truly Yours,

J. W. H. LOVEJOY,

Chairman Com. on Transportation.

To aid the work of the Committee on Transportation, the State Department of our Government at Washington has kindly instructed the resident U. S. Consuls at European ports from which the steamships leave, to actively aid in ascertaining the number of those wishing to avail themselves of the reduced rates offered.

